# Mucins and associated O-glycans based immunoprofile for stratification of colorectal polyps: clinical implication for improved colon surveillance

**DOI:** 10.18632/oncotarget.12347

**Published:** 2016-09-29

**Authors:** Shiv Ram Krishn, Sukhwinder Kaur, Yuri M. Sheinin, Lynette M. Smith, Shailendra K. Gautam, Asish Patel, Maneesh Jain, Vasthala Juvvigunta, Priya Pai, Audrey J. Lazenby, Hemant K. Roy, Surinder K. Batra

**Affiliations:** ^1^ Department of Biochemistry and Molecular Biology, University of Nebraska Medical Center, Omaha, Nebraska, USA; ^2^ Department of Pathology and Microbiology, University of Nebraska Medical Center, Omaha, Nebraska, USA; ^3^ Department of Biostatistics, College of Public Health, University of Nebraska Medical Center, Omaha, Nebraska, USA; ^4^ Department of Surgery, University of Nebraska Medical Center, Omaha, Nebraska, USA; ^5^ Department of Internal Medicine, University of Nebraska Medical Center, Omaha, Nebraska, USA; ^6^ Fred and Pamela Buffett Cancer Center, Eppley Institute for Research in Cancer and Allied Diseases, University of Nebraska Medical Center, Omaha, Nebraska, USA; ^7^ Department of Gastroenterology, Boston Medical Center, Boston, Massachusetts, USA

**Keywords:** mucins, hyperplastic polyps, sessile serrated adenoma/polyps, tubular adenomas, O-glycans

## Abstract

Sessile serrated adenoma/polyps (SSA/P) are premalignant lesions of colorectal cancer that are difficult to distinguish histologically from hyperplastic polyps (HP) of minimal to no malignant potential. Specific markers for differentiating SSA/P from HP can aid clinicians for optimizing colon surveillance intervals. The present study investigates the potential of mucins and associated O-glycans to distinguish SSA/P from HP. Expression of colonic mucins (MUC1, MUC4, MUC17, MUC2, and MUC5AC) and O-glycans [Sialyl Lewis^A^ (CA19-9) and Tn/Sialyl-Tn on MUC1] were analyzed in HP (*n*=33), SSA/P (*n*=39), and tubular adenoma (TA) (*n*=36) samples by immunohistochemistry. A significantly reduced expression of MUC4 (*p*=0.0066), elevated expression of MUC17 (*p*=0.0002), and MUC5AC (*p*<0.0001) was observed in SSA/P cases in comparison to HP cases. Interestingly, significantly higher number of SSA/P cases (*p*<0.0001) exhibited MUC5AC expression in the goblet cells as well as filled the crypt lumen compared to only goblet cells in majority of the HP cases. Improved diagnostic potential was revealed by multivariate logistic regression analysis where combinatorial panel of MUC5AC/MUC17 discriminated SSA/P from HP (SN/SP=85/82%). Finally, the decision tree model based marker panel (CA19-9/MUC17/MUC5AC) predicted HP, SSA/P and TA with SN/SP of 58%/95%, 79%/90% and 97%/83%, respectively. Overall, the mucin and associated O-glycan based panel defined in the present study could aid in discriminating SSA/P from HP to devise better colon surveillance strategies.

## INTRODUCTION

Colorectal cancer (CRC) is a molecularly and histologically heterogeneous, biologically diverse public health problem with over one million new cases diagnosed worldwide every year [1]. The multistep process of colorectal carcinogenesis is associated with the accumulation of genetic and epigenetic alterations in epithelial cells *via* distinct molecular pathways including the most common “adenoma-carcinoma” pathway and the recently identified “serrated polyp neoplasia” pathway [2–4]. Conventional adenomas [tubular adenoma (TA), villous adenoma (VA), and tubulovillous adenoma (TVA)] exhibit characteristic cytological dysplasia and are widely regarded as premalignant lesions accounting for 65-70% of CRCs [5]. In contrast to this, the serrated neoplasia pathway is associated with serrated polyps and accounts for 15-30% of CRCs, and potentially the majority of the interval cancers [6, 7]. According to the latest World Health Organization criteria, the heterogeneous group of serrated lesions is pathologically classified as hyperplastic polyps (HP), sessile serrated adenoma/polyps (SSA/P) and traditional serrated adenomas (TSA) [8]. Traditionally, the small HP found mainly in the distal colon are considered to be benign and non-neoplastic [9], while the larger sessile serrated polyps found mainly in the proximal colon are associated with synchronous and metachronous advanced adenocarcinomas [10, 11]. Epidemiology studies of large cohorts suggest that a stepwise progression of dysplasia and carcinoma from SSA/P may take 10 to 15 years [12] however; there are case reports that suggest faster neoplastic progression [13] or progression taking place in as little as eight months [10, 14, 15].

The differential risk of HP and SSA/P for malignant progression mandates accurate discrimination of these premalignant lesions for improved screening and surveillance. However, due to high cost and low specificity, testing of genetic events like KRAS and BRAF mutations is impractical [16], while histologic evaluation is often compromised because of shared morphological features. As a result, a diagnostic “gray zone” exists between HP and SSA/P, with a wide interobserver and biomarker variability [17–19]. As current surveillance recommendations are dictated by the histologic type of the polyps, therefore, inaccurate classification of these premalignant precursors may lead to an inappropriate follow-up that may potentially contribute to interval carcinomas or unnecessary intervention [20]. Therefore, sensitive and specific markers that could effectively distinguish SSA/P from other polyp subtypes are urgently needed.

Secreted and transmembrane mucins are the most abundant components and major building blocks of the dynamic stratified gastrointestinal mucus layer including that of the colon [21–23]. Alterations in mucins and their associated carbohydrate structures play a role in various gastrointestinal disorders including inflammatory bowel diseases and CRC [24]. Recent analyses of a combinatorial panel of apomucin backbones and associated O-glycans by our group demonstrated their potential to discriminate premalignant and malignant lesions of the colon from normal colon, inflamed colon and benign HP [22]. Further, a recent study identified the transmembrane mucin MUC17 as a potential immunohistochemical marker for SSA/P [25]. In another study, Bartley *et al.* found the relatively high specificity (82%) but low sensitivity (54%) of MUC6 for SSA/P [26], while Renauld *et al.* observed MUC5AC hypomethylation as an early marker of polyps with malignant potential [27]. However, most of these studies were conducted on single or few mucins, and did not consider alterations in mucin-associated O-glycans. Further, there is no study that investigated mucins and associated O-glycans as a combinatorial biomarker panel for the stratification of polyp subtypes. Considering the high clinical utility of the appropriate polyp classification, in this study, we evaluated the potential of colonic mucins (MUC1, MUC4, MUC17, MUC2, MUC5AC, and MUC6) and associated glycans (Tn/STn-MUC1 and CA19-9) for differentiating SSA/P from HP and TA.

## RESULTS

Our earlier findings indicated a differential expression of mucins and their associated O-glycans during the progression of CRC and we demonstrated their utility in differentiating benign lesions from precancerous (polyps with malignant potential-SSA/P and TA) and cancerous ones [22]. Owing to the lack of reliable markers for distinguishing SSA/P from other polyp subtypes, we studied the expression of transmembrane mucins (MUC1, MUC4, MUC17), secreted mucins (MUC2, MUC5AC, MUC6), and O-glycans (Tn/STn-MUC1 and CA19-9) to identify a panel of markers that could efficiently discriminate SSA/P from other polyp subtypes. The clinical and endoscopic findings of the patients and their polyp samples included in this study were retrieved from the electronic medical records. These are summarized in Table 1. There were no significant differences among polyps with regard to age, sex ratio, ethnicity, family history of colon cancer, tobacco consumption, polyp size, and prior or subsequent colonoscopies (Table 1).

**Table 1:** Demographic and clinicopathological characteristics of patients included in the study

		HP	SSA/P	TA	*p*-value
		(*n*= 33)	(*n*= 39)	(*n*= 36)	
**Gender**	Female	17 (52%)	22 (56%)	18 (50%)	0.93
	Male	11 (33%)	15 (39%)	14 (39%)	
	Unknown	5 (15%)	2 (5%)	4 (11%)	
**Age**	Median (Range)	59 (50-91)	60 (28-77)	61 (47-84)	0.9
**Ethnicity**	African American	6 (18%)	2 (5%)	3 (8%)	0.19
	Caucasian	20 (61%)	30 (77%)	24 (67%)	
	Hispanic	0 (0%)	0 (0%)	1 (3%)	
	Asian	0 (0%)	1 (3%)	0 (0%)	
	Unknown	7 (21%)	6 (15%)	8 (22%)	
**Polyp site**	Cecum	0 (0%)	4 (10%)	4 (11%)	**0.025**
	Ascending	2 (6%)	10 (26%)	10 (28%)	
	Transverse	1 (3%)	6 (15%)	6 (17%)	
	Descending	4 (12%)	5 (13%)	3 (8%)	
	Sigmoid	10 (30%)	7 (18%)	4 (11%)	
	Rectum	9 (27%)	5 (13%)	5 (14%)	
	Unknown	7 (21%)	2 (5%)	4 (11%)	
**Family History of colon cancer**	No	18 (55%)	29 (74%)	27 (75%)	0.34
	Yes	7 (21%)	6 (15%)	4 (11%)	
	Unknown	8 (24%)	4 (10%)	5 (14%)	
**Tobacco**	No	6 (18%)	13 (33%)	10 (28%)	0.5
	Yes	20 (61%)	22 (57%)	21 (58%)	
	Unknown	7 (21%)	4 (10%)	5 (14%)	
**Polyp size (cm**^3^)	Mean (SD)	0.048 (0.084)	0.101 (0.168)	0.088 (0.174)	0.41
**Number of polyps**	Median (range)	2 (1-7)	2 (1-7)	3 (1-7)	**0.018**
**Prior or subsequent colonoscopies with polyps**	Median (range)	1 (1-3)	3 (0-5)	1 (0-3)	0.28

HP, hyperplastic polyp; SSA/Ps, sessile serrated adenoma/polyp; TA, tubular adenoma

### Association of mucins and associated O-glycans expression in colorectal polyps with clinicopathological features

As summarized in Table 2, expression of mucins and associated O-glycans was independent of age, gender, family history of colon cancer, and polyp size of the patient. Interestingly, expression of MUC5AC (*p* = 0.027) and CA19-9 (*p* = 0.021) was significantly higher in the polyps from Caucasian patients in comparison to those from other ethnicities. Of all mucins examined, the expression of MUC4 was significantly altered by polyp site (*p* = 0.037) and tobacco consumption (*p* = 0.021). Expression of MUC4 was higher in polyps resected from descending colon compared to all other sites and in polyps from patients with a history of tobacco use. Levels of MUC1 were significantly high (*p* = 0.034) in cases having ≥ 2 polyps in number compared to < 2 polyps. MUC2 levels were significantly high (*p* = 0.024) in the polyps of individuals who had undergone ≥ 2 prior or subsequent colonoscopies.

**Table 2:** Association of mucins and associated O-glycans expression with clinicopathological characteristics

		*N*	MUC1	MUC4	MUC17	MUC2	MUC5AC	CA19-9	Tn/STn-MUC1
			Mean ± SE	Mean ± SE	Mean ± SE	Mean ± SE	Mean ± SE	Mean ± SE	Mean ± SE
**Age**	< 60 years	47	0.77 ± 0.09	1.31 ± 0.11	0.5 ± 0.07	1.71 ± 0.11	0.88 ± 0.09	1.21 ± 0.15	1.34 ± 0.07
	≥60 years	50	0.72 ± 0.08	1.27 ± 0.11	0.64± 0.06	1.8 ± 0.1	0.68 ± 0.09	0.98 ± 0.14	1.2 ± 0.07
	*p*-value		0.76	0.79	0.093	0.54	0.1	0.25	0.37
**Gender**	Female	57	0.74 ± 0.07	1.29 ± 0.1	0.57 ± 0.06	1.7 ± 0.1	0.71 ± 0.08	1.12 ± 0.14	1.28 ± 0.06
	Male	40	0.75 ± 0.1	1.29 ± 0.12	0.58 ± 0.08	1.84 ± 0.12	0.87 ± 0.11	1.04 ± 0.15	1.25 ± 0.09
	*p*-value		0.67	0.96	0.68	0.25	0.32	0.41	0.61
**Ethnicity**	Caucasian	74	0.73 ± 0.06	1.31 ± 0.09	0.56 ± 0.05	1.73 ± 0.09	0.83 ± 0.08	1.18 ± 0.12	1.22 ± 0.06
	Other	13	0.87 ± 0.18	1.31 ± .21	0.48 ± 0.12	1.85 ± 0.17	0.4 ± 0.12	0.45 ± 0.21	1.45 ± 0.15
	*p*-value		0.51	0.95	0.58	0.79	**0.027**	**0.021**	0.15
**Polyp site**	Cecum	8	0.65 ± 0.1	1.03 ± .14	0.75 ± 0.1	1.6 ± 0.16	0.75 ± 0.15	1.19 ± 0.24	1.15 ± 0.1
	Ascending	22	0.73 ± 0.12	1.28 ± .22	0.56 ± 0.19	1.96 ± 0.12	0.93 ± 0.24	1.01 ± 0.28	1.28 ± 0.15
	Transverse	13	0.5 ± 0.15	1.11 ± 0.27	0.55 ± 0.16	1.43 ± 0.32	0.54 ± 0.25	0.84 ± 0.36	1.05 ± 0.14
	Descending	12	0.68 ± 0.17	1.73 ± 0.19	0.55 ± 0.1	1.57 ± 0.19	0.59 ± 0.09	0.95 ± 0.21	1.35 ± 0.13
	Sigmoid	21	0.74 ± 0.12	1.42 ± 0.16	0.43 ± 0.09	1.98 ± 0.15	0.79 ± 0.11	1.17 ± 0.22	1.3 ± 0.11
	Rectum	19	1.02 ± 0.15	0.93 ± 0.2	0.59 ± 0.11	1.88 ± 0.19	1.06 ± 0.24	1.03 ± 0.28	1.39 ± 0.13
	*p*-value		0.16	**0.037**	0.33	0.33	0.47	0.93	0.42
**FH of colon cancer**	no	74	0.77 ± 0.07	1.27 ± 0.09	0.56 ± 0.05	1.72 ± 0.09	0.77 ± 0.08	0.97 ± 0.11	1.28 ± 0.06
	yes	17	0.62 ± 0.09	1.38 ± 0.14	0.49 ± 0.1	1.92 ± 0.11	0.76 ± 0.18	1.44 ± 0.25	1.13 ± 0.1
	*p*-value		0.58	0.66	0.64	0.65	0.9	0.064	0.15
**Tobacco**	no	29	0.63 ± 0.09	1.02 ± 0.13	0.51 ± 0.07	1.65 ± 0.15	0.89 ± 0.14	1.1 ± 0.18	1.28 ± 0.1
	yes	63	0.79 ± 0.08	1.43 ± 0.1	0.56 ± 0.06	1.8 ± 0.09	0.7 ± 0.08	1.02 ± 0.13	1.24 ± 0.06
	*p*-value		0.3	**0.021**	0.78	0.52	0.27	0.51	0.84
**Polyp size**	<0.05 cm^3^	66	0.7 ± 0.07	1.3 ± 0.09	0.53 ± 0.06	1.78 ± 0.08	0.72 ± 0.08	0.98 ± 0.12	1.25 ± 0.07
	≥0.05 cm^3^	24	0.83 ± 0.14	1.31 ± 0.16	0.68 ± 0.09	1.75 ± 0.17	0.98 ± 0.13	1.29 ± 0.21	1.32 ± 0.08
	*p*-value		0.51	0.9	0.11	0.84	0.087	0.17	0.27
**Number of polyps**	<2	21	0.58 ± 0.14	1.3 ± 0.17	0.62 ± 0.11	1.91 ± 0.12	0.97 ± 0.17	1.28 ± 0.22	1.33 ± 0.13
	≥2	75	0.79 ± 0.06	1.29 ±0.09	0.57 ± 0.05	1.71 ± 0.09	0.72 ± 0.07	1.02 ± 0.12	1.25 ± 0.06
	*p*-value		**0.034**	0.99	0.76	0.44	0.2	0.35	0.41
**Prior or subsequent** **colonoscopies with polyps**	<2	45	0.7 ± 0.08	1.05 ± 0.13	0.6 ± 0.08	1.48 ± 0.11	0.71 ± 0.1	0.99 ± 0.15	1.19 ± 0.07
	≥2	21	0.69 ± 0.11	1.25 ± 0.14	0.6 ± 0.1	1.91 ± 0.14	0.84 ± 0.15	0.95 ± 0.2	1.35 ± 0.11
	*p*-value		0.94	0.31	0.78	**0.024**	0.48	0.83	0.25

FH, family history; SE, standard error

### Expression of mucins and associated O-glycans in colorectal polyps and adjacent normal colon mucosa

The expression of MUC1, MUC5AC, Tn/STn-MUC1, and CA19-9 were significantly increased in all types of polyp lesions (*p* < 0.0005) compared to adjacent normal colon (NC) (Table 3). Expression of MUC17 was significantly high in HP (*p* < 0.0005) and SSA/P (*p* < 0.0005) with statistically non-significant alterations in TA compared to NC (Table 3). Transmembrane mucin MUC4 expression was significantly decreased in SSA/P (*p* < 0.0005) with statistically non-significant alterations in HP and TA compared to NC (Table 3). In comparison to NC, the expression of secretory mucin MUC2 was significantly decreased in both SSA/P (*p* < 0.005) and TA (*p* < 0.0005) and non-significantly altered in HP (Table 3).

**Table 3:** Expression of mucins and associated O-glycans in different colorectal polyp subtypes and corresponding adjacent normal colon

		NC	HP	NC	SSA/P	NC	TA
**MUC1**	Mean ± SE	0.25±0.07 ^e^	0.76 ± 0.09 ^b^	0.09±0.02 ^l^	0.53 ± 0.09 ^c,h^	0.28±0.05 ^i^	1.02 ± 0.09 ^c,k^
	Median (Min, Max)	0.1(0,1.4)	0.6(0.1, 2.1)	0.05(0, 0.3)	0.4(0.05, 2.4)	0.2(0, 1)	1(0.1, 2.4)
**MUC4**	Mean ± SE	1.87±0.11	1.66 ± 0.12 ^k^	1.63±0.15 ^l^	1.05 ± 0.12 ^c,e^	1.25±0.12	1.21 ± 0.12
	Median (Min, Max)	2.1(0.2, 2.55)	1.8(0.3, 2.7)	1.8(0.1, 2.4)	1(0.05, 2.7)	1.2(0.05, 2.4)	1.3(0.1, 2.4)
**MUC17**	Mean ± SE	0.11±0.03 ^f^	0.37 ± 0.05 ^c,l^	0.06±0.02 ^l^	0.85 ± 0.08 ^c,f,g^	0.24±0.05	0.47 ± 0.07 ^j^
	Median (Min, Max)	0.05(0, 0.7)	0.2(0.05, 1.2)	0(0, 0.3)	1(0.05, 2.1)	0.1(0, 1.2)	0.4(0, 1.4)
**MUC2**	Mean ± SE	2.31±0.09	2.09 ± 0.09 ^h^	2.26±0.09 ^k^	1.92 ± 0.08 ^b,g^	2.21±0.06 ^i^	1.35 ± 0.14 ^c,e,j^
	Median (Min, Max)	2.4(1.2, 2.7)	2.4(0.75, 2.7)	2.4(0.9, 2.7)	1.8(1.2, 2.7)	2.4(1, 2.7)	1.3(0.1, 2.7)
**MUC5AC**	Mean ± SE	0±0 ^f^	0.65 ± 0.08 ^c,l,i^	0±0 ^l^	1.24 ± 0.09 ^c,f,i^	0.03±0.02 ^i^	0.3 ± 0.08 ^c,f,l^
	Median (Min, Max)	0(0, 0)	0.6(0.15, 2.1)	0(0, 0)	1.2(0.15, 2.4)	0(0, 0.6)	0.06(0, 2.1)
**Tn/STn-MUC1**	Mean ± SE	0.23±0.05 ^f^	1.42 ± 0.07 ^c^	0.17±0.05 ^l^	1.39 ± 0.08 ^c^	0.39±0.05 ^i^	1.13 ± 0.1 ^c^
	Median (Min, Max)	0.1(0, 0.8)	1.4(0.6, 2.7)	0(0, 0.8)	1.4(0.3, 2.4)	0.3(0, 1.6)	1.2(0, 2.4)
**CA19-9**	Mean ± SE	0.1±0.05 ^f^	1.53 ± 0.17 ^c,i^	0.04±0.02 ^l^	1.72 ± 0.13 ^c,i^	0.02±0.01 ^i^	0.1 ± 0.03 ^c,f,l^
	Median (Min, Max)	0(0, 1.2)	1.8(0, 2.7)	0(0, 0.4)	2.1(0, 2.7)	0(0, 0.3)	0(0, 0.6)

^a^*p* < 0.05 *vs*. adjacent normal; ^b^*p* < 0.005 *vs*. adjacent normal; ^c^*p* < 0.0005 *vs*. adjacent normal; ^d^*p* < 0.05 *vs*. HP; ^e^*p* < 0.005 *vs*. HP; ^f^*p* < 0.0005 *vs*. HP; ^g^*p* < 0.05 *vs*. TA; ^h^*p* < 0.005 *vs*. TA; ^i^*p* < 0.0005 *vs*. TA; ^j^*p* < 0.05 *vs*. SSA/P; ^k^*p* < 0.005 *vs*. SSA/P; ^l^*p* < 0.0005 *vs*. SSA/P

NC, adjacent normal colon; HP, hyperplastic polyps; SSA/P, sessile serrated adenoma/polyps; TA, tubular adenoma; SE, standard error; Min, minimum; Max, maximum

### Differential expression of mucins and associated O-glycans among colorectal polyp subtypes

Looking at the aberrant expression of mucins and associated O-glycans in different colorectal polyps compared to NC, we next investigated whether mucins and associated O-glycans are differentially expressed between benign (HP) and pre-cancerous lesions of the colon (SSA/P and TA).

#### SSA/P *vs*. HP

**Figure 1:**
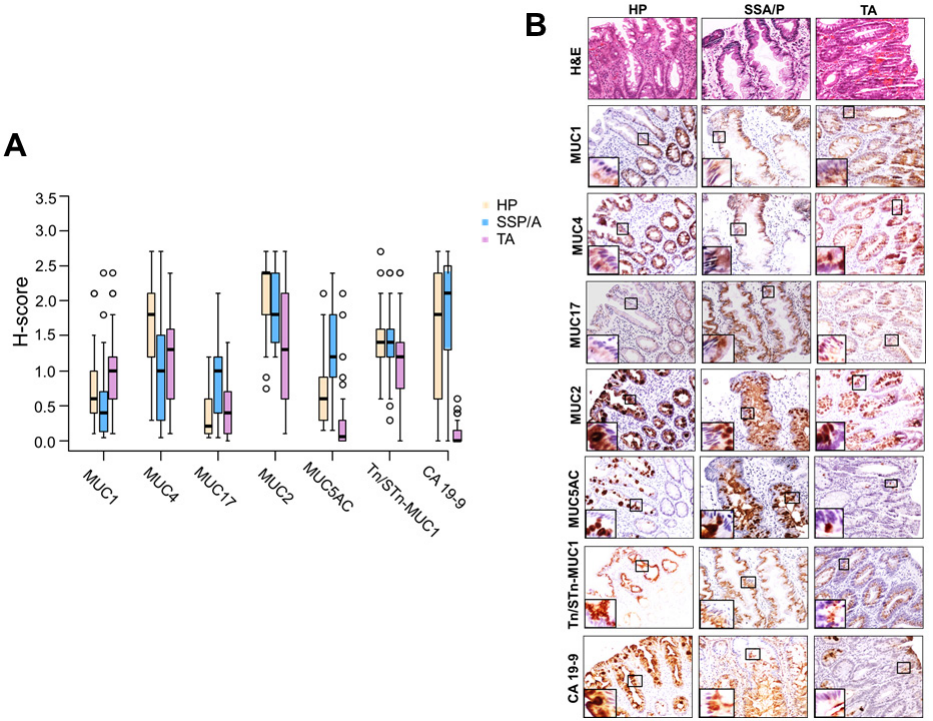
Immunohistochemical expression of mucins and associated O-glycans in colorectal polyps **A**. Box plots representing the H-score for mucins (MUC1, MUC4, MUC17, MUC2, and MUC5AC) and O-glycans (CA19-9, Tn/STn-MUC1) expression in HP, SSA/P and TA. A significant downregulation of MUC4 (*p* = 0.0066), significant upregulation of MUC17 (*p* = 0.0002) and MUC5AC (*p* < 0.0001) was observed in SSA/P cases in comparison to HP cases. The box plots further show that compared to SSA/P in TA cases, MUC1 expression was significantly increased (*p* = 0.0005) while there was a significant decrease in expression of MUC17 (*p* = 0.0054), MUC2 (*p* = 0.02), MUC5AC (*p* < 0.0001) and CA19-9 (*p* < 0.0001). **B**. The HP, SSA/P, and TA tissue specimens were probed with MUC1 (mouse monoclonal-HMFG2), MUC4 (mouse monoclonal-8G7), MUC17 (rabbit polyclonal-SN-1139-2), MUC2 (rabbit monoclonal-EPR6145), MUC5AC (mouse monoclonal-45M1), CA19-9 (mouse monoclonal-NS 19.9), and Tn/STn-MUC1 (mouse monoclonal-5E5) antibodies after non-specific blocking with horse serum. All the tissue sections were examined under a microscope, and the expression was evaluated on basis of reddish brown staining by a pathologist. Representative photomicrographs (20X) of Hematoxylin & Eosin staining, mucins (MUC1, MUC4, MUC17, MUC2, and MUC5AC), and O-glycans (Tn/STn-MUC1 and CA19-9) stained tissues of HP, SSA/Ps, and TA were taken. For uniformity, images for all the mucins and O-glycans staining were taken from the same area in a sample of HP, SSA/P and TA respectively. Magnified images in inset shows expression of respective mucins in different cellular compartments. MUC1 expression was significantly high (*p* = 0.0005) in TA in comparison to HP and SSA/P. Strong expression of MUC4 was observed in adjacent normal colon. Expression of MUC4 was significantly reduced in SSA/P (*p* = 0.0066) compared with HP. Significantly high immunoreactivity of MUC17 was observed in SSA/P lesions compared to HP and TA. Compared to HP and SSA/P, MUC2 expression was significantly reduced in TA (*p* = 0.0018, and 0.020 respectively). MUC5AC expression was significantly high in HP and SSA/P compared to TA (*p* < 0.0001). HP, SSA/P, and TA expressed Tn/STn-MUC1 at similar levels. CA19-9 expression was significantly high in HP (*p* < 0.0001) and SSA/P (*p* < 0.0001) compared to TA. NC: adjacent normal colon; HP: hyperplastic polyps; SSA/P: sessile serrated adenoma/polyps; TA: tubular adenoma.

In comparison to HP, SSA/P exhibited reduced expression of MUC4 (*p* = 0.0066), elevated expression of MUC17 (*p* = 0.0002) and MUC5AC (*p* < 0.0001) (Figure 1A, 1B; Table 3). Interestingly, in SSA/P, the strong reactivity of MUC4 (H-Score > 2) was restricted to 10% of cases in comparison to the 45% of HP cases. While moderate to the strong reactivity (H-Score > 1) of MUC17, MUC5AC was observed in 33% and 62% of SSA/P cases, respectively, compared to only 6% and 15% of HP cases respectively.

#### SSA/P *vs*. TA

TA exhibited elevated expression of MUC1 (*p* = 0.0005) and decreased expression of MUC17 (p = 0.0054), MUC2 (*p* = 0.02), MUC5AC (*p* < 0.0001), and CA19-9 (*p* < 0.0001) in comparison to SSA/P (Figure 1A, 1B; Table 3). Moderate to strong reactivity (H-Score > 1) of MUC1 was observed in 47% of TA compared to 13% of SSA/P. Further, moderate to strong reactivity (H-Score > 1) of MUC17, MUC2, MUC5AC, and CA19-9 was observed in a greater proportion of SSA/P cases (33%, 100%, 62%, and 82% respectively) compared to the TA cases (11%, 58%, 8%, and 0% respectively).

### Alteration in mucins and associated O-glycans localization

Interestingly, mucins and associated O-glycans exhibited a distinct expression pattern in HP, SSA/P, TA lesions in comparison to adjacent normal colon. In both adjacent normal colon and polyp subtypes, cytoplasmic expression of MUC17 and Tn/STn-MUC1 was observed in crypt epithelial cells. CA19-9 expression was absent in adjacent normal colon while in the polyp lesions its expression was observed to be in goblet cells and cytoplasm of crypt epithelial cells. In adjacent normal colon, MUC2 expression was observed solely in the goblet cells, while in the polyp lesions, it was expressed in both the goblet cells as well as the in cytoplasm of crypt epithelial cells. MUC1 was uniformly expressed in the cytoplasm of the crypt epithelial cells in adjacent normal colon as well as in polyp lesions.

**Figure 2:**
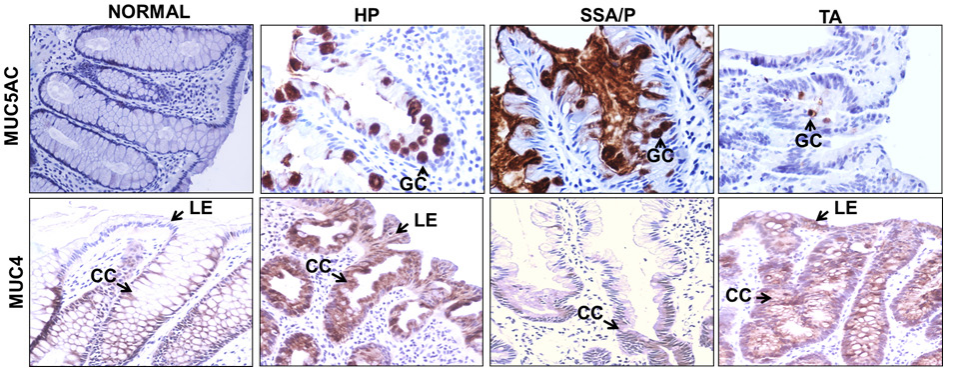
Differential localization of mucins in colorectal polyp subtypes Differential localization pattern for MUC5AC and MUC4 were observed for polyp subtypes. MUC5AC expression was absent in normal colon, restricted to the goblet cells (GC) in HP, and present in the goblet cells and lumen of colon crypts in SSA/P. In case of TA, the MUC5AC expression was observed in goblet cells but at lower intensity. In normal colon, MUC4 expression was observed throughout the colon crypts (CC) while it was absent on luminal surface epithelium (LE). In the HP and TA, MUC4 expression was observed throughout CC including luminal epithelium. In contrast to this, in 30% SSA/P cases, MUC4 expression was limited to lower one-third of colon crypts. The differential localization pattern of MUC4 and MUC5AC could provide useful molecular adjunct to histological classification of colorectal polyps. CC: colon crypts; GC: goblet cells; LE: luminal surface epithelium; HP: hyperplastic polyps; SSA/P: sessile serrated adenoma/polyps; TA: tubular adenoma.

While secretory mucin MUC5AC expression was undetectable in the adjacent normal colon, it was overexpressed in all HP and SSA/P cases. Notably, a stark difference in the MUC5AC expression pattern was observed within the colon crypts among the study groups. In HP, MUC5AC expression was limited to goblet cells in 61% cases, while the remaining 39% exhibited overexpression in the goblet cells as well as hypersecretion in lumen of the colon crypts. In contrast, in 89% SSA/P cases (*p* < 0.0001) significantly increased MUC5AC expression was observed in both goblet cells and secreted in the lumen of colon crypts (Figure 2) while in the remaining 11% it was restricted to goblet cells only. Unlike MUC5AC, the normal colon expressed MUC4, which was localized in cytoplasmic compartments throughout the crypt epithelial cells but absent from luminal surface epithelium. In HP and TA, expression of MUC4 was observed throughout the crypt as well as on luminal surface epithelium. Interestingly, in approximately 30% SSA/P cases, MUC4 expression was limited to lower one-third of the crypts only, while in the remaining 70%, its expression was similar to HP and TA (Figure 2).

### Mucins and associated O-glycans as potential molecular marker(s) for differentiating colon polyp subtypes

Considering the significant differences in the levels and expression patterns of several mucins and associated O-glycans, we next evaluated their individual or combinatorial utility to discriminate the polyp-subtypes. To investigate this, we utilized the univariate logistic regression analysis along with receiver-operator characteristics (ROC) curves analysis.

#### SSA/P *vs*. HP

Low expression of MUC4 and increased expression of MUC17 and MUC5AC significantly differentiated SSA/P polyps from HP with the sensitivity and specificity (SN/SP) of 61.5%/70%, 67%/91%, and 77%/73% respectively (Supplementary Figure 1A; Table 4).

**Table 4:** Univariate and Multivariate logistic regression and ROC curve analysis of mucins and associated O-glycans for colorectal polyps classification

Univariate Analysis													
Marker	Diagnosis		OR (95% CI)		*p*-value		AUC (95% CI)		Cutpoint		Sensitivity		Specificity
**MUC1**	SSA/P *vs*. HP		0.411 (0.156, 1.084)		0.072		0.6772 (0.5531, 0.8012)		<=0.30		0.487		0.848
	SSA/P *vs*. TA		0.188 (0.069, 0.509)		0.001		0.7639 (0.6545, 0.8733)		<=0.60		0.744		0.722
**MUC4**	SSA/P *vs*. HP		0.324 (0.160, 0.657)		0.0018		0.7257 (0.6089, 0.8425)		<=1.2		0.615		0.697
	SSA/P *vs*. TA		0.745 (0.395, 1.405)		0.36		0.5630 (0.4319, 0.6942)		<=1.2		0.615		0.5
**MUC17**	SSA/P *vs*. HP		14.376 (3.664, 56.41)		0.0001		0.7739 (0.6623, 0.8855)		>=0.8		0.667		0.909
	SSA/P *vs*. TA		5.990 (1.955, 18.346)		0.0017		0.7222 (0.6047, 0.8397)		>=0.80		0.667		0.778
**MUC2**	SSA/P *vs*. HP		0.530 (0.210, 1.337)		0.18		0.5960 (0.4640, 0.7279)		<=1.8		0.513		0.727
	SSA/P *vs*. TA		3.288 (1.573, 6.873)		0.0016		0.6991 (0.5765, 0.8216)		>=1.60		0.718		0.583
**MUC5AC**	SSA/P *vs*. HP		9.112 (2.912, 28.51)		0.0001		0.7995 (0.6947, 0.9043)		>=0.9		0.769		0.727
	SSA/P *vs*. TA		21.755 (5.985, 79.078)		<0.0001		0.9081 (0.8320, 0.9842)		>=0.60		0.923		0.806
**Tn/STn-MUC1**	SSA/P *vs*. HP		0.870 (0.317, 2.390)		0.79		0.5085 (0.3756, 0.6415)		<=0.8		0.179		0.939
	SSA/P *vs*. TA		2.472 (1.016, 6.016)		0.046		0.6353 (0.5095, 0.7611)		>=1.2		0.795		0.444
**CA19-9**	SSA/P *vs*. HP		1.279 (0.746, 2.194)		0.37		0.5287 (0.3907, 0.6668)		>2.1		0.333		0.606
	SSA/P *vs*. TA		54.383 (6.489, 455.7)		0.0002		0.9551 (0.9095, 1.0000)		>=0.80		0.872		1
**Multivariate Analysis**													
**Diagnosis**		**Comparison**	**AUC**	**Lower**		**Upper**		**Threshold**		**Sensitivity**		**Specificity**	
**SSA/P** *vs***. HP**		MUC17+MUC5AC	0.865	0.776		0.954		P(SP)>= 0.4928		0.846		0.818	
**SSA/P** *vs***. TA**		MUC1+MUC17+CA19-9	0.9868	0.97		1		P(SPT)>=0.385		0.923		0.972	

HP, hyperplastic polyps; SSA/P, sessile serrated adenoma/polyps; TA, tubular adenoma; OR, odds ratio; AUC, area under curve; CI, confidence interval

#### SSA/P *vs*. TA

Low expression of MUC1, higher levels of MUC17, MUC2, MUC5AC, Tn/STn-MUC1, and CA19-9 significantly differentiated SSA/P from TA with the sensitivity and specificity (SN/SP) of 74.4%/72.2%, 67%/78%, 72%/58.3%, 92.3%/81%, 79.5%/44.4%, and 87.2%/100% respectively (Supplementary Figure 1B; Table 4).

### Combinatorial panels of mucins and associated O-glycans for differentiating colorectal polyp sub-types

Looking at some of the individual markers that exhibited better sensitivity while others exhibited better specificity to differentiate colorectal polyp subtypes, we evaluated the combined utility of significantly altered mucins and O-glycans as markers for differential identification of polyp subtypes. For this, multivariate regression models were created using the most promising markers from the univariate logistic regression analysis, in conjunction with ROC curve based analysis.

#### SSA/P *vs*. HP

**Figure 3:**
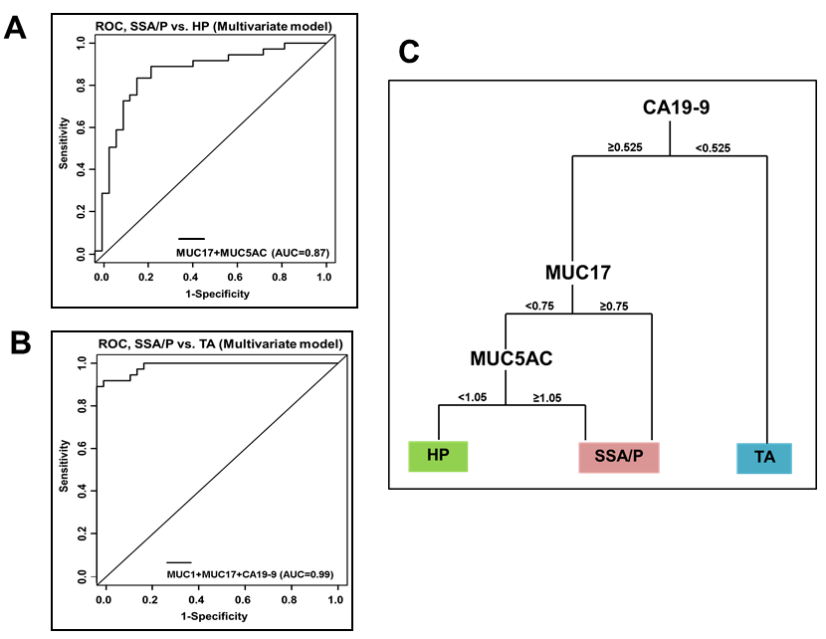
Combined performance of mucins and associated O-glycans for discriminating colorectal polyp subtypes The combined utility of significantly altered mucins and O-glycans as markers for differential identification of polyp subtypes was evaluated utilizing multivariate regression models in conjunction with ROC curve based analysis. **A**. ROC curves representing diagnostic potential of combinatorial panels of mucins and associated O-glycans for differentiating SSA/P from HP. MUC17+MUC5AC combination differentiated SSA/P from HP with the AUC of 0.87. The model has 83% accuracy (60/72) in predicting SSA/P *vs*. HP. **B**. MUC1 and MUC17+CA19-9 combination differentiated SSA/P from TA with the AUC of 0.99. the model has 95% accuracy (71/75) in predicting SSA/P *vs*. TA. **C**. Decision tree based model to accurately define the combined diagnostic efficacy of mucins and associated O-glycans for polyp stratification. The panel of three markers CA19-9/MUC17/MUC5AC differentiated precancerous polyps (SSA/P and TA) from benign polyps (HP). Polyp sections having H-Score < 0.525 for CA19-9 predicts the presence of TA. For tissues sections having H-Score ≥ 0.525 for CA19-9, and H-score ≥ 0.75 for transmembrane mucin MUC17 the lesion is likely to be SSA/P. In cases where MUC17 H-score is < 0.75, and MUC5AC H-score is ≥ 1.05, then SSA/P is predicted and if MUC5AC H-score < 1.05, then HP is predicted. The tree based model has 79% accuracy (85/108) in predicting all 3 groups simultaneously.HP: hyperplastic polyps; SSA/P: sessile serrated adenoma/polyps; TA: tubular adenoma.

The combination of MUC17/MUC5AC effectively discriminated SSA/P from HP with SN/SP of 85%/82% (Figure 3A; Table 4). The optimal cut point value based on the H-Score derived from the model [P(SSA/P) ≥ 0.49] suggests that, if the predicted probability from the combined MUC17/MUC5AC model is ≥ 0.49 then the model predicts SSA/P, and if it is < 0.49 then model predicts HP. The model has 83% accuracy (60/72) in predicting SSA/P *vs*. HP.

#### SSA/P *vs*. TA

The combination of MUC1/MUC17/CA19-9 effectively discriminated SSA/P from TA with SN/SP of 92.3%/97.2% (Figure 3B; Table 4). Further, optimal cutpoint [P(SSA/P) ≥ 0.385] derived from the model suggests that if the predicted probability from the combined MUC1/MUC17/CA19-9 model is ≥ 0.385 then the model predicts SSA/P and if it is < 0.385 then model predicts TA. The model has 95% accuracy (71/75) in predicting SSA/P *vs*. TA.

#### CA19-9/MUC17/MUC5AC: a novel biomarker combination for differentiating polyp subtypes

Next, we evaluated clinical utility of the significantly varying markers obtained from multivariate logistic regression results utilizing the tree based modeling to predict the diagnostic power for differentiating various lesions (HP, SSA/P, or TA) (Figure 3C). The combined utility of three markers (CA19-9, MUC17 and MUC5AC in order of preference) was identified for differential diagnosis of precancerous polyps (polyps with malignant potential-SSA/P and TA) from benign polyps (HP) (Figure 3C). According to this model, if the observed CA19-9 H-Score is < 0.525, the polyp under observation is predicted to be TA. If CA19-9 H-Score is ≥ 0.525 then the next marker considered is MUC17 (Figure 3C). If MUC17 H-Score is ≥ 0.75, lesion is likely to be SSA/P. However, if MUC17 H-Score is < 0.75, then next marker considered is MUC5AC. If H-Score of MUC5AC is ≥ 1.05, then SSA/P is predicted and if MUC5AC H-Score < 1.05, then HP is predicted (Figure 3C). Overall, the tree based model predicted HP, SSA/P and TA with SN/SP of 58%/95%, 79%/90% and 97%/83% respectively. The tree based model has 79% accuracy (85/108) in predicting all 3 groups simultaneously. Thus, we demonstrated the utility of differentially expressed mucins and associated O-glycans for effective discrimination of colorectal polyp subtypes at histological level.

## DISCUSSION

Colonoscopy has been a gold standard colon screening option. However, a substantial increase in CRCs arising during intervals between surveillance colonoscopy has been reported [28]. Interestingly, the precursor lesions for these interval cancers were hypothesized to be SSA/Ps. Despite association of serrated polyps with CRC [11], the identification for serrated polyps at the morphological, histopathological, and molecular levels remains challenging. This confusion and inconsistency translates in misdiagnoses of serrated lesions due to interobserver variability and ultimately inappropriate follow-up care and therapy. Studies have demonstrated differential expression of mucins in colorectal polyp subtypes. Unfortunately, most of these studies were either limited to one or few mucins, did not consider modifications of associated O-glycan alterations, and focused only on detecting expression levels without emphasizing diagnostic utility.In addition, the previous studies did not explore the combinatorial power of mucins and associated O-glycans. We recently demonstrated the combined utility of mucins and associated O-glycans as markers to discriminate benign polyps from premalignant and malignant lesions [22]. Building on these observations the current study for the first time investigated expression of the major mucins and associated O-glycans that are significantly altered during colon carcinogenesis for discriminating SSA/P from HP and TA.

Previous studies have suggested that MUC1 is either absent or is weakly expressed in the normal colon [29, 30]. Focal to mild reactivity in HP and its inability to discriminate them from SSA/P has previously been described [29, 31–33]. In contrast, increased MUC1 positivity has been reported in conventional adenomas [29]. Consistent with these studies, we observed focal to mild immunoreactivity of MUC1 in SSA/P and significantly higher expression in TA. Based on these observations and a previous report demonstrating MUC1 expression to be independent of microsatellite status [34], it can be suggested that MUC1 may not be a prominent player in the serrated pathway of neoplasia. However, higher levels of MUC1 in TA along with reports of its even higher expression in CRC [30], and association with invasion risk [33] argue for the potential applicability of MUC1 expression as a predictor of malignant transformation in the adenoma-carcinoma sequence.

Congruent with previous studies [29], MUC4 appeared to be a prominent mucin gene expressed in the normal colon. Loss of MUC4 in CRC cases has previously been demonstrated [22, 35]. Interestingly, we observed a significant loss of MUC4 in SSA/P compared to HP, suggesting that loss of MUC4 might play an important role during serrated pathway of neoplasia. MUC4 was differentially expressed in SSA/P and TA *versus* benign lesions (HP) however; it did not appear as a significant contributor in the combinatorial panels of markers that discriminated these polyp subtypes. Interestingly, MUC4 was the only mucin whose expression was significantly impacted by the site of the polyp, suggesting the role of colonic microenvironment in the regulation of this mucin. Interestingly, we also found that MUC4 expression was significantly higher in polyps of the patients with a history of tobacco use. Long-term cigarette smoking is associated with colorectal polyps and increased risk of CRC mortality [36, 37]. Recently, smoke exposure was reported to result in increased Muc4 expression in the distal colon of mice [38]. Similarly, reports have shown that cigarette smoke upregulates expression of MUC4 [39]. The presence of MUC4 confers worse prognosis to early stage CRC patients [35, 40]. Therefore, in future it would be interesting to investigate the role of cigarette smoke-mediated upregulation of MUC4 during CRC progression.

MUC17 expression is significantly increased in CRC cases compared to colon inflammation cases [22]. In this study, we not only conducted comprehensive analysis of MUC17 expression in different polyp subtypes, but also observed its exceptional utility as a biomarker to discriminate SSA/P from HP and TA. MUC17 has previously been shown to be a top differentially expressed gene in SSA/P *versus* TA [41]. RNA-seq analysis also indicated 82-fold increase in MUC17 in SSA/P compared to adenomatous polyps, uninvolved and normal colon samples [25]. Interestingly, none of these studies considered HP to compare differential expression of genes in SSA/P, thus limiting the overall interpretations in terms of molecular characterization of SSA/P for their efficient diagnosis. We observed significantly increased MUC17 expression in HP and SSA/P compared to NC. Most importantly, MUC17 was significantly overexpressed in SSA/P in comparison to HP and TA cases, and emerged as a significant independent marker of SSA/P *versus* HP and TA with high specificity. In multivariate models, the combination of MUC17 and MUC5AC improved the diagnostic accuracy of SSA/P *versus* HP with 85% sensitivity and 82% specificity. Furthermore, MUC17 was found to be a significant contributor to the panel of diagnostic markers that discriminated SSA/P from TA. Due to the paucity of information regarding the association of MUC17 with BRAF mutation, CpG island methylator phenotype (CIMP), microsatellite instability (MSI) and microsatellite instability-high (MSI-H) colon carcinomas, additional studies addressing these aspects would help establish MUC17 as a marker of neo-transformation through the serrated neoplasia pathway.

Several studies have previously reported MUC5AC expression in normal colon, HP, SSA/P, TSA, TA, VA, TVA and mixed polyps [29, 30, 42–44] without emphasizing on its diagnostic utility for discriminating these polyp subtypes. Consistent with these studies, we also observed significant alterations in the expression of MUC5AC in HP, SSA/P, and TA when compared to the NC. Further, significant overexpression of MUC5AC was observed in SSA/P when compared to HP and TA. Conceptually, high positivity of MUC5AC represents an early gain of gastric differentiation in SSA/P and is significantly associated with MSI-H CRCs sporadic tumors (*p* < 0.001) [45]. Increased expression of MUC5AC in SSA/P has recently been attributed to MUC5AC hypomethylation which in turn, is specifically associated with serrated lesions with BRAF mutation, CIMP-H or MSI [27]. Therefore, increased expression of MUC5AC in SSA/P may be reflective of neoplastic process. Interestingly, combination of MUC5AC and MUC17 discriminated SSA/P from HP with an area under curve (AUC) of 0.865 and SN/SP of 86%/82%. Moreover, MUC5AC was the only marker which could significantly differentiate SSA/P from HP and TA on basis of its differential localization pattern. Given these results, IHC for these markers may be helpful to pathologists for effective discrimination of SSA/P from benign HP. Studies characterizing the role of MUC5AC during the serrated pathway of CRC progression would establish its utility as a predictive marker of neoplastic transformation.

Increased expression of MUC6 was found to be highly specific in distinguishing SSA/P from HP [16, 26]. Contrary to this, it was also reported that increased expression of MUC6 lacks specificity in distinguishing SSA/P and SSA/P with dysplasia from HP [31]. Owens *et al*. concluded that neither anatomic location nor polyp size account for the differences in MUC6 expression in serrated polyps [16, 42]. However, Bartley *et al*. concluded strong association of MUC6 expression with proximal location of serrated polyps [26]. Looking at these confounding reports, we also investigated expression of MUC6 in SSA/P, TA and HP samples. However, MUC6 immunoreactivity was absent in majority of cases (data not shown). These observation conflicts from previous studies could be attributed to difference in tissue sites, patient ethnicity, antibody used for study and/or limited sample numbers.

CA19-9 is a monosialosyl Lewis (a) blood group antigen that has been shown to be differentially expressed during adenoma-carcinoma sequence [22]. The importance of CA19-9 serum levels at the beginning of malignant alterations for monitoring colorectal tumors [46] and to discriminate premalignant lesions from cancer has previously been reported [47]. This is the first study to evaluate CA19-9 in SSA/P and assess its efficacy to discriminate colorectal polyps in combination with other mucins. The present study clearly demonstrated that CA19-9 expression is significantly increased in all colorectal polyp subtypes with highest levels in SSA/P, and lowest in TA in comparison to HP. Diagnostic utility of CA19-9 was evident from the fact that CA19-9 in combination with MUC1 and MUC17 predicted SSA/P from TA with very high sensitivity and specificity (92.3%/97.2%). Our assessment from multivariate models was further corroborated by a decision tree model where CA19-9 proved to be an effective marker to clearly discriminate TA from SSA/P and HP at the very first step.

Further, this study utilized, the ‘Tree-based models’ as an additional exploratory way of analyzing the data. Such models are advantageous in the sense that interactions are handled automatically as well as monotonic transformations of the x and y variables. They also easily handle multiple categories for the classification variable. However, ‘Tree based models’ are usually preferred when sample sizes are large. For our case, since the sample size was relatively small, we tested accuracy using both multivariate and tree-based model.

The promising results from this study lay the foundation for stratification of colorectal polyp subtypes on the basis of mucins and associated O-glycans immunostaining. The proposed panel of markers from this study, in conjunction with current histologic diagnostic criterion may aid pathologists in better discrimination of the colorectal polyp subtypes. However, this study is limited by its small sample size and lack of enough samples from patients who had multiple polyps from different sites or of different subtypes. Further, Torlakovic *et al*. histologically subclassified HPs on basis of the epithelial mucin content into goblet cell-rich, microvesicular, and mucin-poor types [48]. Therefore, future studies could focus on further investigating the mucins and associated O-glycans expression in various subtypes of HP for further improvement in discrimination from SSA/P.

Cancer-associated post-translational modifications frequently produce aberrant O-linked glycoproteins which in combination with protein backbone induce auto-antibodies [49]. One recent study reported cancer-associated autoantibodies against a set of aberrantly glycosylated glycopeptides derived from mucin MUC1 and MUC4 in sera of CRC patients [49]. Therefore, future research efforts should also focus attention on identification of CRC-specific O-glycans on differentially expressed mucins (MUC1, MUC2, MUC4, MUC5AC, and MUC17) and develop CRC-specific glycopeptide microarrays for serum profiling of patients harboring colorectal polyps with malignant potential.

Efforts should continue to further elucidate the currently unexplored roles of mucins MUC4, MUC17, and MUC5AC in the progression of CRC *via* different pathways, along with their association with various genetic and epigenetic mutations in CRC. This will firmly establish their utility as markers for molecular classification of polyps. Also, investigation of molecular mechanisms associated with the upregulation of mucins like MUC17 and MUC5AC in SSA/P may further help in devising therapies targeting these lesions.

In conclusions, this study is the first critical step addressing the “gray zone” in differentiating SSA/P from other polyp subtypes utilizing aberrant mucin expression and glycosylation. The efficient discrimination of different colorectal polyp subtypes utilizing the novel panel of molecular markers (CA19-9/MUC17/MUC5AC) from the decision tree model, in conjunction with currently available tests and colonoscopy, may aid clinicians in devising improved colon cancer screening recommendations.

## MATERIALS AND METHODS

### Tissue specimens

After institutional review board (IRB) approval (IRB#167-15-EP), endoscopically resected formalin-fixed paraffin embedded colonic polyp tissue specimens- HP (*n* = 33), SSA/P (*n* = 39), and TA (*n* = 36) were obtained from the Department of Pathology at University of Nebraska Medical Center (UNMC). The polyp diagnoses were based on the current WHO criteria [8]. Additionally, only those cases were considered for the study where the original histological diagnosis by pathologists was in consensus to the diagnosis made by a second pathologist (Dr. Yuri Sheinin at UNMC). The colorectal polyps used for this study includes samples from the same patient having different subtypes and/or multiple polyps of one type from different sites in colon. However, their numbers were insignificant. The normal colon tissue adjacent to the polyp lesion was also determined and considered for comparative analysis by the pathologist (Dr. Yuri M. Sheinin) on the basis of histological examination of Hematoxylin & Eosin stained tissue sections.

### Immunohistochemistry (IHC)

IHC was performed on the polyps following a standard protocol [50]. After overnight baking at 56°C, tissue sections were deparaffinized with xylene (10 min, 4X), rehydrated with graded alcohol (5 minutes each wash). Endogenous peroxidase activity was quenched using methanolic 3% hydrogen peroxide (30 minutes). Antigen was retrieved in 0.05M citrate buffer (pH 6.0, 95°C, 15 minutes), and blocked with 2.5% horse serum (ImmPRESS Universal antibody kit; Vector Laboratories, Burlingame, CA) (two hours). Tissue sections were then incubated with anti-mucin and associated O-glycans specific antibodies overnight at 4°C (Supplementary Table 1). Next, the tissue sections were washed with PBS-T (10 minutes, 4X) and incubated with anti-rabbit/anti-mouse secondary antibody conjugated with Horseradish peroxidase (ImmPRESS Universal antibody kit; Vector Laboratories) (30 minutes). Next, the sections were washed with PBS-T (10 minutes, 4X), developed for colorimetric detection by 3, 3’ diaminobenzidine kit (Vector Laboratories, Burlingame, CA, USA) and counterstained with hematoxylin. Tissues were dehydrated (graded alcohol and xylene), dried, mounted with Permount, and evaluated by the pathologists.

The staining scores corresponding to the polyps as well as the normal colon adjacent to polyps was determined following the H-Score system published previously [51]. The intensity of staining for each of the markers was scored on a scale of 0-3 (0-negative, 1-weak, 2-intermediate, 3-strong intensity) to obtain intensity score (IS). For a given intensity, the percentage of cells positive for mucin or associated O-glycans within a given lesion was also scored on a scale of 0-1 (0: no cells staining, 1: 100% cells positively stained). The IS and corresponding score for the percentage of immunoreactive cells were then multiplied to obtain a histology Score (H-Score), representing overall expression ranging from 0-3. The respective tissue specimens were further classified by H-score as having no reactivity (H-score = 0), focal reactivity (H-score > 0 but ≤ 0.1), mild reactivity (H-score > 0.1 but ≤ 1), moderate reactivity (H-score > 1.0 but ≤ 2.0) and strong reactivity (H-score > 2.0). Further, differential localization pattern of mucin and associated O-glycans expression within colon crypts was also considered for analysis.

### Statistical analysis

Patient characteristics for each polyp subtype were compared using chi-square or Fisher's exact test for categorical variables and ANOVA or Kruskal-Wallis test for continuous variables. H-score for each polyp subgroup were compared using Kruskal-Wallis test and Wilcoxon rank sum test for pairwise comparisons. Wilcoxon signed rank test was used to compare H-Score of polyp subtypes and the respective adjacent normal controls. Adjustments for multiple comparisons were made using Bonferroni's method. H-score was also categorized into reactivity groups, and these were compared by polyp subtype using Chi-square test. The localization pattern of secretory mucin MUC5AC in HP and SSA/P crypts was compared using Chi-square test. Univariate and multivariate logistic regression models and ROC curves were used to examine the individual markers (MUC1, MUC4, MUC17, MUC2, MUC5AC, CA19-9 and Tn/STn-MUC1), and a combinatorial panel of markers as predictors of each polyp subtype. ROC curves were used to determine optimal H-score cutpoints for markers. Firth's penalized maximum likelihood estimation was used to reduce bias in the parameter estimates because of separability in some of the multivariate models. Accuracy of multivariate models to differentiate SSA/P from TA and HP was calculated as the number of cases classified correctly divided by the total number of cases considered. SAS software version 9.3 was used for all other statistical analyses (SAS Institute Inc., Cary, NC). Further, the R package rpart was used to create a classification tree useful for biomarker-based medical decision making. Accuracy of tree based model was calculated as the number of cases classified correctly divided by the total number of cases considered.

## SUPPLEMENTARY MATERIALS FIGURES AND TABLES



## Abbreviations

AUC: area under curve
CI: confidence interval
CRC: colorectal cancer
H-Score: histology- score
HP: hyperplastic polyps
IS: intensity score
MUC: mucin
NC: adjacent normal colon
OR: odds ratio
ROC: receiver operating characteristics
SE: standard error
SN: sensitivity
SP: specificity
SSA/P: sessile serrated adenoma/polyps
TA: tubular adenoma
TSA: traditional serrated adenoma
TVA: tubulovillous adenoma
VA: villous adenoma
