# Supplementary material for: Mucins and associated O-glycans based immunoprofile for stratification of colorectal polyps: clinical implication for improved colon surveillance
Source: Oncotarget. 2016 Sep 29;8(4):7025–38. doi: 10.18632/oncotarget.12347 (PMC5351688; doi:10.18632/oncotarget.12347)
Supplement: Supplementary file 1 [file oncotarget-08-7025-s001.pdf]

Mucins and associated O-glycans based immunoprofile for stratification of colorectal polyps: clinical implication for improved colon surveillance

Supplementary Material

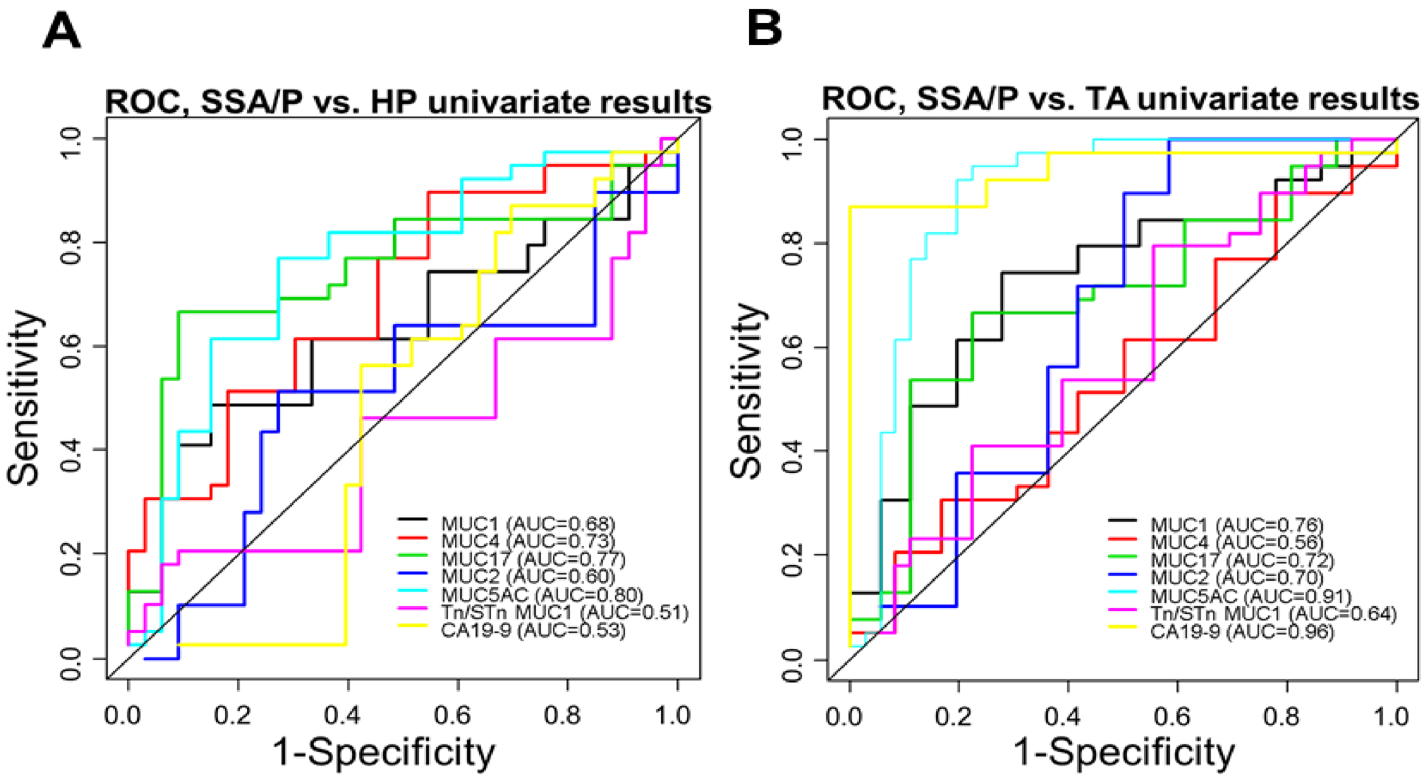

Supplementary Figure 1. Univariate and ROC curve analysis comparing different mucins and associated O-glycans individually for discriminating colorectal polyp subtypes. (A) MUC4, MUC17, and MUC5AC individually were the significant predictors of SSA/P versus HP. **(B)** MUC1, MUC17, MUC2, MUC5AC, Tn/STn-MUC1, and CA19-9 individually were the significant predictors of SSA/P versus TA.

HP: hyperplastic polyps; SSA/P: sessile serrated adenoma/polyps; TA: tubular adenoma

Supplementary Table 1: Panel of antibodies used for IHC analysis of various mucins and associated O-glycans

S.no.	Parameter	Antibody	Origin and type	Source/Reference
1.	MUC1	HMFG2	Mouse monoclonal	[52]
2.	MUC4	8G7	Mouse monoclonal	[53]
3.	MUC17	SN-1139-2	Rabbit polyclonal	[54]
4.	MUC2	EPR6145	Rabbit monoclonal	Abcam (ab134119)
5.	MUC5AC	45M1	Mouse monoclonal	Abcam (ab3649)
6.	MUC6	CLH5	Mouse monoclonal	Abcam (ab49462)
7.	CA 19-9	1116-NS-19-9	Mouse monoclonal	Thermo Fisher Scientific (MA1-34608)
8.	Tn/STn on MUC1	5E5	Mouse monoclonal	[55]
